# Effect of Lipid Emulsion on Stability of Ampicillin in Total Parenteral Nutrition

**DOI:** 10.3390/nu11030559

**Published:** 2019-03-06

**Authors:** Maciej Stawny, Aleksandra Gostyńska, Katarzyna Dettlaff, Anna Jelińska, Eliza Główka, Magdalena Ogrodowczyk

**Affiliations:** 1 Department of Pharmaceutical Chemistry, Poznan University of Medical Sciences, 6 Grunwaldzka, 60-780 Poznań, Poland; aleksandra.gostynska@spsk2.pl (A.G.); dettlaff@ump.edu.pl (K.D.); ajelinsk@ump.edu.pl (A.J.); mogrodo@ump.edu.pl (M.O.); 2 Department of Pharmaceutical Technology, Poznan University of Medical Sciences, 6 Grunwaldzka, 60-780 Poznań, Poland; eglowka@ump.edu.pl

**Keywords:** total parenteral nutrition, ampicillin, physicochemical stability, lipid emulsion

## Abstract

Background: Ampicillin (AMP) is frequently administered parenterally in critically ill patients with meningitis or endocarditis. Many of them require parallel infusion of total parenteral nutrition (TPN) admixtures. The aim of the study was to determine the physicochemical stability of AMP in TPN admixtures. Methods: AMP was added to two formulations of TPN admixtures differing in the lipid emulsion (Lipofundin^®^ MCT/LCT 20% or LIPIDem^®^). Samples were stored at 4 ± 1 °C with light protection, and at 25 ± 1 °C with and without light protection to assess the impact of temperature and light on formulation stability. Every 24 h the pH, zeta potential, mean droplet diameter (MDD) of a lipid emulsion, and AMP concentration using HPLC method were determined. The assessment of stability and compatibility of TPN admixtures with vitamins and trace elements was carried out immediately after preparation and after 24 h of storage. Results: The addition of AMP as well as vitamins and trace elements to the TPN admixtures did not affect their physical stability. An increase in the pH value of approx. 0.6 and reduction of zeta potential were observed. The MDD of the lipid emulsions was below the limit of 500 nm (dynamic light scattering (DLS) method) and no fat droplets greater than 525 nm were observed (light diffraction (LD) method). The content of AMP after the first 24 h was within the acceptable limit of 90% for TPN admixtures stored at 4 ± 1 °C and 25 ± 1 °C with light protection. Conclusions: The results showed that co-administration of AMP in the same bag with TPN admixture at the tested dose is possible when used ex tempore and with light protection.

## 1. Introduction

Ampicillin (AMP) is a β-lactam antibiotic with a wide range of activity often used in clinical practice. High doses of AMP are frequently administered parenterally in critically ill patients with meningitis or endocarditis. Such patients often receive concomitantly an intravenous infusion of total parenteral nutrition (TPN) admixtures in order to maintain a proper nutritional status and to alleviate the metabolic response resulting from stress, injury, or extensive surgery. In some cases, co-administration of drugs in the same infusion with TPN is needed due to a limitation of available intravenous (IV) lines. There are few reports in the literature regarding physicochemical compatibility and stability of TPN admixtures and IV drugs. However, in recent years this topic has attracted more attention [1,2,3,4,5]. Administration of a drug with unknown stability is a direct threat to a patient’s health and life. It is particularly important to confirm the possibility of using medications in parallel with other medicines via Y-site or adding them to a medium not described in the SPC (Summaries of Product Characteristics) [6]. TPN admixture is a compounded drug resulting from the combination of amino acid, glucose, electrolyte, and lipid emulsion. The determination of an IV medication’s stability in this kind of medium is very problematic and requires appropriate physicochemical assessment proving stability and compatibility of both TPN admixtures and the added drug [7,8,9].

In this study, physical stability studies of TPN admixtures were conducted to determine the effect of AMP, time, and storage conditions on formulation stability. For this purpose, we assessed such physical parameters as pH, mean droplet diameter (MDD) of the lipid emulsions, and zeta potential of TPN admixtures with and without AMP. Regarding the chemical stability of AMP, high-performance liquid chromatography (HPLC) was performed.

To the best of our knowledge, so far there have been no studies confirming the possibility of adding AMP to TPN admixtures in adults. Therefore, we decided to investigate this scenario. The aim of the study was to determine the physicochemical compatibility and stability of AMP in two standard TPN admixtures containing different types of lipid emulsion, which is key to ensure patients’ safety.

## 2. Materials and Methods

### 2.1. Compositions of TPN Admixtures

In this study, we chose two compositions of TPN admixtures differing in the type of lipid source (Table 1). TPN admixtures composition was based on the literature recommendations for adult hospitalized patients and calculated for a patient weighing 60 kg [10]. The only difference between studied compositions was the type of lipid emulsion. Lipofundin^®^ MCT/LCT-containing TPN admixtures (A, A + AMP) consisted of a lipid emulsion based on long-chain (LCT) and medium-chain (MCT) triglycerides. In contrast, the LIPIDem^®^-containing TPN admixtures (B, B + AMP) were based on a lipid emulsion consisting of MCT, LCT, and triglycerides of omega-3 acids. For a 7-day stability study, we prepared TPN admixtures without vitamins and trace elements, as in clinical practice these components, due to their low stability, are added just before administration to patients. Therefore, the physical stability studies of TPN admixtures (A, A + AMP, B, and B + AMP) with vitamins (one vial of Cernevit, Baxter SA, Lessines, Belgium) and trace elements (one ampoule of Tracutil, B. Braun Melsungen AG, Germany) were carried out only on the day of preparation of the TPN admixtures and after 24 h.

### 2.2. TPN Admixtures and Samples Preparation

The TPN admixtures were prepared in aseptic conditions under a laminar-flow air hood using a Pinnacle B. Braun automatic compounder (B. Braun Melsungen AG, Germany). All doses were calculated as one-tenth of standard daily dose. The final volume of 250 mL was packaged in an ethylene-vinyl acetate (EVA) bag with a capacity of 300 mL (B. Braun Melsungen AG, Germany).

For sample preparation, a dose of 1 g of AMP was chosen as one-tenth of the standard dose used for serious bacterial infections like bacterial meningitis or endocarditis. The maximum dose of AMP for adult patients is 14 g per day. The tested materials were TPN admixtures mixed with 1 g of AMP (Ampicillin TZF 1000 mg) previously dissolved in 10 mL of water for injection (A + AMP, B + AMP) and reference samples were TPN admixtures without the addition of the drug (A, B). Each admixture was prepared separately in triplicate.

The A + AMP admixtures were stored at 4 ± 1 °C with light protection, and at 25 ± 1 °C with and without light protection. The other TPN admixtures (A, B, and B + AMP) were stored at 4 ± 1 °C with light protection. Physicochemical analysis was performed on the day of admixture preparation and after 24 h, 48 h, 72 h, 92 h, 120 h, and 144 h. Samples were aseptically collected from each TPN admixture at appropriate intervals using a plastic syringe for the physicochemical tests. Every 24 h, a volume of 10.0 mL was withdrawn from each TPN admixture and divided into two samples. For chemical stability tests, 3.0 mL was used, and the rest was used for physical stability assays (visual inspection, pH, mean droplet diameter, and zeta potential determination).

**Figure 1:**
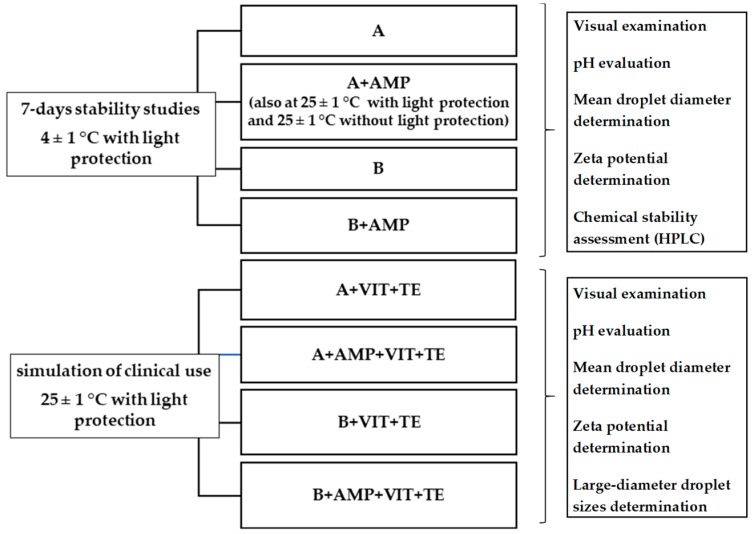
Diagram of study design. TPN: total parenteral nutrition; A: Lipofundin^®^ MCT/LCT-containing TPN admixture without ampicillin; B: LIPIDem^®^-containing TPN admixture without ampicillin; A + AMP, B + AMP: TPN admixtures with ampicillin; A + VIT + TE, B + VIT + TE: TPN admixtures with vitamins and trace elements; A + AMP + VIT + TE, B + AMP + VIT + TE: TPN admixtures with ampicillin, vitamins, and trace elements.

Additionally, for the tested TPN admixtures (A, B, A + AMP, B + AMP) with vitamins and trace elements, the distribution of large-diameter droplet sizes assessment was performed in accordance with the requirements of the US Pharmacopoeia [11]. The measurements were taken immediately after the preparation of the samples and after 24 h. A routine gravity infusion set (Exadrop, B. Braun AG Melsungen) was used for sample collection, a procedure simulating the administration of TPN admixtures to hospital patients. All samples were stored at 25 ± 1 °C with light protection. A diagram of the study design is presented in Figure 1. 

### 2.3. Physical Stability

#### 2.3.1. Visual Examination

In accordance with the European Pharmacopoeia [12], all samples were visually assessed for the presence of particles and color change. Visual inspection was performed against a black-and-white contrast background by two observers.

#### 2.3.2. pH Evaluation

For the evaluation of pH, a Mettler Toledo Seven Compact pH/ion S220^®^ pH-meter was used. The pH was measured by dipping the electrode directly into the TPN admixture, at room temperature. The pH of each sample was measured in triplicate.

#### 2.3.3. Mean Droplet Diameter and Zeta Potential Determination

The intensity weighted mean droplet diameter (MDD) of lipid emulsion, polydispersity index (PDI), and zeta potential of TPN admixtures were measured at 25 °C using a Zetasizer Nano ZS (Malvern Instruments, Malvern, UK) by dynamic light scattering (DLS) and laser Doppler velocimetry (LDV), respectively. A dip cell (zen1002, Malvern Instruments) with a pair of parallel Pd electrodes was used to provide electrical trigger on charged particles. The signals were collected at an angle of 12.8° and the data were analyzed using Zetasizer Software. As suggested by ISO13099 [13,14], the Smoluchowski model was used to calculate zeta potential values of nanoparticles in aqueous media. Samples for size and zeta potential measurement were prepared in accordance to the same protocol. One milliliter of tested TPN admixtures or reference samples was diluted 10 times with water for injection and then 1 mL of diluted sample was transferred to a Malvern Clear Zeta Potential cell for DLS and LDV measurements.

#### 2.3.4. Large-Diameter Droplet Sizes Determination

The percent of particles below 500 nm, 525 nm, and above 5 µm were estimated by light diffraction (LD) method based on Mie theory using Mastersizer^®^ 3000 analyzer (Malvern Panalytical, Malvern, UK). The Hydro SV small volume wet sample dispersion unit was used and set at 1500 rpm. The analyzer typically enables particle size distribution from 10 nm to 3500 µm to be measured depending on sample and sample preparation. The cuvette was filled with degassed distilled water passed through a filter with 0.2 µm porosity and the background was measured. Undiluted samples (15 µL) were added to the cuvette until obscuration was achieved in the range of 1% to 5%. The refractive and absorption indices were set at 1.449 and 0.01, respectively. Each sample was measured in triplicate.

### 2.4. Chemical Stability Assessment

An HPLC method was used for the quantitative determination of AMP in the TPN admixtures. The Merck Hitachi L-7100 HPLC system was used. The analytical column was a 250 mm × 4.6 mm in diameter reverse phase C18 (LiChrospher^®^ 100, endcapped, 5 µm) coupled with 4-mm length pre-column reverse phase C18 (LiChrospher^®^ 100, 5 µm). As mobile phase solvent A (12% acetic acid 0.5 mL, 0.2 M potassium dihydrogenphosphate 50 mL, acetonitrile 50 mL, water ad 1000 mL) and solvent B (12% acetic acid 0.5 mL, 0.2 M potassium dihydrogenphosphate 50 mL, acetonitrile 400 mL, water ad 1000 mL) at the ratio 95:5 (*v*/*v*) were used. Separation was performed at 25 °C, the mobile phase flow was 1 mL/min, and the analytical wavelength was 230 nm. Samples for HPLC analysis were prepared by mixing 3.0 mL of TPN admixture with 1.0 mL of chloroform in 10-mL plastic vials. The vials were shaken for 10 min and then centrifuged for 15 min at a rate of 5800 rpm. Samples were collected from the centrifuged supernatant, filtered through a membrane filter (pore size 0.2 µm), and injected into a chromatographic column for HPLC analysis (sample volume 10 µL).

### 2.5. Statistical Analysis

The data were analyzed using Statistica 12 software (StatSoft Polska, Cracow, Poland). Repeated-measures analyses of variance (ANOVAs) were used to determine whether the drug content, storage conditions, or storage time had an effect on the pH, the MDD of lipid emulsion, and the zeta potential. The a priori level of significance was *p* < 0.05. In the case of a major effect or interaction, significant differences between the tested TPN admixtures (with drugs) and the reference samples under the study conditions were identified using Tukey’s honest significant difference test post hoc tests. The significance was defined as *p* < 0.05.

## 3. Results

### 3.1. Physical Stability

**Figure 2:**
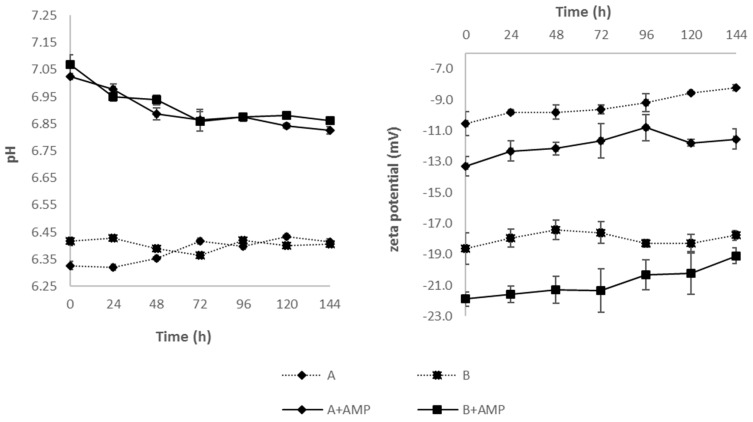
Results of pH and zeta potential of TPN admixtures without (A, B) and with (A + AMP, B + AMP) ampicillin during storage at 4 ± 1 °C with light protection. Results are shown as the mean values of three samples ± SD.

**Figure 3:**
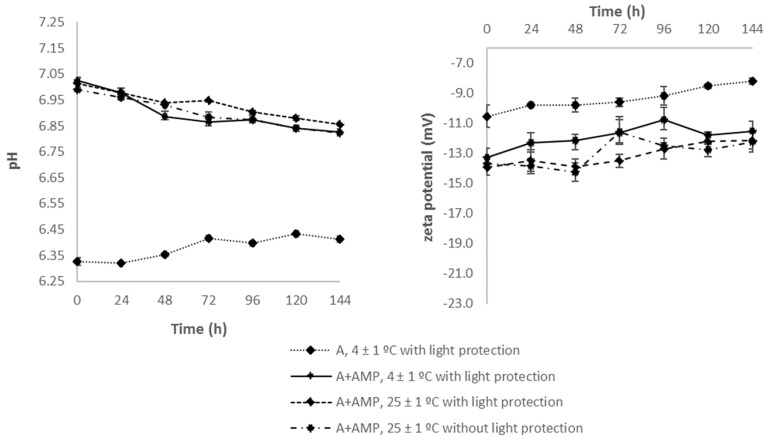
Values of pH and zeta potential of studied basic composition of TPN admixtures with (A + AMP) and without AMP (A) during storage at different conditions. Results are shown as the mean values of three samples ± SD.

During the entire study, none of the tested samples showed the presence of particles or color change. Immediately after preparation (t = 0 h), the measured pH values of reference samples (A, B) were 6.33 ± 0.02 and 6.42 ± 0.02 for Lipofundin^®^ MCT/LCT-containing and LIPIDem^®^-containing TPN admixtures, respectively (Figure 2). The addition of AMP increased significantly (*p* < 0.05) the pH by approx. 0.6 in both formulations (A + AMP, B + AMP). The pH values of Lipofundin^®^ MCT/LCT-containing TPN admixtures with AMP decreased slightly but significantly in time regardless of the storage conditions (Figure 3). The same pattern was observed in the case of LIPIDem^®^-containing TPN admixtures with AMP (Figure 2).

Analyzing the results of the MDD of lipid emulsions in TPN admixtures, there was no statistically significant differences (*p* > 0.05) observed between TPN admixtures without AMP (A, B) and corresponding samples containing AMP (A + AMP, B + AMP) stored at 4 ± 1 °C with light protection (Table 2). MDDs of the lipid emulsions in TPN admixtures without AMP (A, B) varied from 220.2 ± 5.4 nm to 228.2 ± 0.9 nm for Lipofundin^®^ MCT/LCT-containing TPN admixtures, and 219.1 ± 5.6 nm to 227.2 ± 1.0 nm when LIPIDem^®^ was used. The results showed that the lipid source was not associated with MDD values. The addition of AMP to the TPN admixtures did not cause any significant and characteristic changes in the MDD of the lipid emulsion particles, which remained below the limit of 500 nm. The storage conditions also did not affect the MDD of the lipid emulsions of Lipofundin^®^ MCT/LCT-containing TPN admixtures (Table 2).

Another important parameter characterizing lipid emulsion particles is the polydispersity index (PDI), which should be in the range 0.05–0.7. Based on the results, it was found that for all tested samples the PDI expressing the degree of inhomogeneity of the system’s particles was in the range from 0.05 to 0.13, so the DLS technique was suitable for measuring the particle size of the studied TPN admixtures.

As shown in Figure 2, the zeta potential values of the reference sample (A, B) on the first day of storage differed significantly between Lipofundin^®^ MCT/LCT-containing (−10.55 ± 0.76 mV) and LIPIDem^®^-containing (−18.63 ± 1.03 mV) TPN admixtures. The addition of AMP resulted in a significant reduction of the zeta potential on the first day of storage (*p* < 0.05). During the study, an increase in the zeta potential was observed. When comparing the results obtained in the following hours to the value of the zeta potential measured immediately after preparation of each sample, no statistically significant differences were found for the reference samples during storage. In the case of samples containing AMP, many results showed statistically significant differences between the zeta potential at t = 0 h (Figure 2 and Figure 3). Interestingly, for samples stored at 25 ± 1 °C with and without light protection, there were almost no differences between zeta potential values obtained on the following day and the value obtained on the first day.

The TPN admixtures containing vitamins and trace elements were slightly yellow, and the color deepened over time. The PDI for Lipofundin^®^ MCT/LCT-containing and LIPIDem^®^-containing TPN admixtures with vitamins and trace elements after preparation was 0.04 and 0.08, respectively. The addition of AMP and 24-h storage at 25 ± 1 °C did not affect PDI, which ranged from 0.07 to 0.09. For samples containing vitamins, trace elements, and AMP, MDDs were slightly lower compared to the corresponding samples without vitamins, trace elements, or AMP (Table 3). In the particle size distributions by volume obtained by laser diffraction method, the values of Dv(90) (90% of the particles lies below the value) were in the range of 446 nm to 457 nm for both fresh formulations and after 24-h storage. All droplet sizes were below 525 nm and there were no particles larger than 5 μm. The addition of vitamins and trace elements caused a slight decrease in pH, whereas the values obtained on the day of preparation were maintained after 24 h. The zeta potential values for the samples containing vitamins and trace elements without and with the addition of AMP were lower than those obtained for analogous samples without these additives (Table 3).

### 3.2. Chemical Stability

AMP concentration after the first 24 h was found to be 92.81 ± 0.56% and 92.97 ± 1.10% of the zero-time concentration for Lipofundin^®^ MCT/LCT-containing and LIPIDem^®^-containing TPN admixtures stored at 4 ± 1 °C (Table 4). Considering Lipofundin^®^ MCT/LCT-containing TPN admixtures with AMP stored at room temperature (at 25 ± 1 °C), our results proved the impact of direct light on accelerated degradation. Samples stored without light protection were unstable even during the first 24 h (89.01 ± 0.85%), whereas those stored at the same temperature with light protection were still within the accepted limit (90.77 ± 1.28%). Chromatographic data showed that prolonged storage of TPN admixtures causes a decrease in the content of AMP above the limit of 10%.

## 4. Discussion

There is lack of consensus on the parameters to be determined when evaluating the physical stability of TPN admixtures. Some authors suggest that the large-diameter droplet tail by single-particle optical sizing technique should be performed to proof absence of particles above 5 µm [15,16,17,18]. However, we chose pH, the particle size of the lipid emulsions by DLS and LD techniques, and the zeta potential. Such methods were used for investigating the stability of TPN admixtures by Garcia et al. [3], Mediavilla et al. [4], and Riera et al. [5]. These parameters can be used for a quick and easy assessment of the stability of lipid emulsions, such as TPN admixtures. According to literature data [15], the risk of destabilization of the oil-water system (TPN admixture) increases when the pH decreases below 5.5. Regarding the particle size changes of lipid emulsions administered intravenously, it should be noted that the largest endogenous lipoproteins, chylomicrons, have a diameter of 75 to 600 nm. Thus, the particle size of lipid emulsions for parenteral administration should not exceed 500 nm, as larger particles could cause capillary blockages and serious clinical consequences, including damage to blood vessels in the lungs, liver, and retina of the eye [16,17]. According to the requirements of the United States Pharmacopoeia, the average particle size of fat emulsions measured by the DLS method must be below 500 nm [11]. It should be emphasized that slight changes in the size of the lipid emulsion particles on consecutive storage days are a common occurrence. The TPN admixture, which is an oil-in-water system, is characterized by the constant movement of lipid emulsion particles, according to Brownian motion. The lack of clear and significant changes in particle size after the addition of AMP indicates that this drug does not significantly affect the stability of the TPN admixture regarding the stability of lipid emulsions. Another parameter characterizing lipid emulsion is zeta potential (i.e., the charge on the surface of lipid emulsion particles), allowing for the assessment of the force of electrostatic interactions between the particles. The zeta potential depends on the concentration of electrolytes and on the pH of the TPN admixture. It has been shown that, due to the presence of stabilizing phospholipids, pharmaceutical formulations of lipid emulsions used for the preparation of TPN admixtures are characterized by zeta potential values from −40 mV to −50 mV, and therefore exhibit considerable stability [19]. It has also been proven that the addition of amino acid solutions to the lipid emulsion reduces the absolute value of the zeta potential by approximately 10 mV [20,21,22]. Moreover, the addition of monovalent sodium, potassium, and bivalent ions of magnesium and calcium significantly reduces the zeta potential through specific and non-specific adsorption on the surface of the emulsion particles. Depending on the composition of TPN admixtures, the zeta potential may have values ranging from −4.1 mV to −1.7 mV, or even close to zero [20,21,22,23]. Such a significant difference in the value of the zeta potential between the LIPIDem^®^-containing and Lipofundin^®^ MCT/LCT-containing TPN admixtures result from the presence of another type of lipid emulsion. LIPIDem^®^ contains 50% medium-chain f triglycerides (MCT), 40% long-chain triglycerides (LCT), and 10% triglycerides of omega-3 acids, whereas Lipofundin^®^ MCT/LCT 20% consists of equal amounts of MCT and LCT. On the basis of the results, it may be suggested that the addition of omega-3 acids stabilizes the oil-water system and increases the absolute value of the zeta potential. Our findings indicate that the addition of AMP causes a reduction in the zeta potential regardless of the type of lipid emulsion. However, after the reduction of zeta potential, a significant increase in its values was observed during storage, thus indicating the destabilizing effect of AMP on lipid emulsion during prolonged storage.

The HPLC method was used to assess the chemical stability of the drug in the TPN admixture. According to the criteria adopted for this study, the content of the drug in TPN should not decrease below 90% of zero-time concentration. Results above the 90% limit were observed for TPN admixtures stored at 4 ± 1 °C and 25 ± 1 °C with light protection but only within the first 24 h. The standard infusion of TPN admixture lasts from 16 to 24 h. Thus, the 24-h chemical stability of AMP in TPN admixture is sufficient for ex tempore administration.

The literature shows conflicting results for AMP compatibility tests, which might be related to the different concentrations of AMP and/or the different ratios and different types of parenteral nutrition admixtures (2-in-1 or total parenteral nutrition) applied in the studies [24]. In the compatibility studies, after Y-site administration of 2-in-1 admixtures (without lipid emulsion) precipitation was observed [1,25]. It was probably the effect of precipitating calcium phosphate when the pH of the TPN admixture increased above 7.2. In our study, the TPN admixtures were prepared using glycerophosphate and calcium gluconate, organic compounds that do not dissociate and do not give free calcium and phosphate ions. For this reason, the precipitation of calcium phosphate or dicalcium phosphate in a mixture of calcium gluconate and glycerophosphate is practically impossible even after exceeding the equilibrium solubility. Moreover, in another study, the administration of 20 mg/mL AMP via Y-site with nine representative TPN admixtures (containing lipid emulsion) at the ratio 1:1 was defined as compatible [26].

The results of our study can be applied both for non-supplemented and supplemented with vitamins and trace elements TPN admixtures as well as for Y-site co-administration of AMP and TPN admixtures. However, the concentration of the drug should be calculated and TPN admixture compositions taken into account. It should be noted that our study was conducted on two types of TPN admixtures differing in the source of lipid emulsion. The composition was based on the ASPEN and ESPEN guidelines. Therefore, the differences in TPN admixture compositions should be considered when evaluating the results of this study. Given that in our study we determined the stability of AMP in a dose of 10 g per day, which is common against meningitis, especially in neurosurgical intensive care units, the possibility of adding other doses to TPN admixtures should be confirmed by chemical stability studies.

## 5. Conclusions

The reference samples of TPN admixtures (A, B) were characterized by adequate stability during storage, regardless of the type of lipid emulsion, temperature, and light access. The observed changes in zeta potential and MDD of lipid emulsions were insignificant and remained within the required values. It was also observed that the addition of AMP to TPN admixture significantly reduced the zeta potential, increased the pH of the TPN admixtures, and slightly changed the MDD of the lipid emulsion. However, all physical stability parameters obtained in this work did not exceed the limits. For all samples (TPN admixtures, TPN admixtures with AMP, TPN admixtures with vitamins and trace elements, and TPN admixtures with vitamins, trace elements, and AMP), the MDD and Dv90 were below 500 nm, which is in line with pharmacopoeial requirements.

Our chemical stability studies indicated that the addition of AMP to TPN admixture is possible only for ex tempore use and with light protection. The decomposition of AMP occurred in both Lipofundin^®^ MCT/LCT-containing and LIPIDem^®^-containing TPN admixtures, indicating that it does not depend on the composition of the fat emulsion but on the temperature and light access during storage.

Our research has demonstrated the importance of evaluating both the physical stability of TPN admixtures and the chemical stability of drugs to be added. The degradation of AMP observed during the days following its addition did not significantly affect the physical stability of the TPN admixture. It may be misleading to rely exclusively on physical stability tests due to the risk of administering a drug with a reduced active substance content and/or harmful degradation products.

## Figures and Tables

**Table 1:** Composition of TPN admixtures.

Ingredients	Pharmaceutical Preparation	Unit	Composition of TPN Admixtures	
			A	B
Amino acid	Aminoplasmal^®^ B. Braun 10% E (B. Braun Melsungen AG, Germany)	mL	600	600
Carbohydrates	40% Glucose B. Braun (B. Braun Melsungen AG, Germany)		550	550
Lipids	Lipofundin^®^ MCT/LCT 20% (B. Braun Melsungen AG, Germany)		300	0
	LIPIDem^®^ (B. Braun Melsungen AG, Germany)		0	300
Water	Aqua ad iniectabile (B. Braun Melsungen AG, Germany)		902	902
Sodium	Natrium Chloratum 10% (B. Braun Melsungen AG, Germany)	mmol	102	102
Potassium	Kalium Chloratum 15% WZF (WZF Polfa S.A., Poland)		80	80
Calcium	Calcium gluconate 10% (Added Pharma, Netherlands)		5	5
Phosphates	Glycophos (Fresenius Kabi AB, Sweden)		24	24
Magnesium	Inj. Magnesii Sulfurici 20% (Polpharma S.A, Poland)		6	6
Total volume		mL	2500	2500
Total energy		kcal	1660	1660

TPN: total parenteral nutrition; A: Lipofundin^®^ MCT/LCT-containing TPN admixture; B: LIPIDem^®^-containing TPN admixture.

**Table 2:** MDD of lipid emulsion in studied TPN admixtures during storage. Results are shown as the mean values of three samples ± SD.

Time (h)	MDD ± SD (nm)					
	A	A + AMP	A + AMP	A + AMP	B	B + AMP
	4 ± 1 °C	4 ± 1 °C	25 ± 1 °C	25 ± 1 °C	4 ± 1 °C	
	With Light Protection	With Light Protection	With Light Protection	Without Light Protection	With Light Protection	With Light Protection
**0**	220.2 ± 5.4	216.9 ± 1.7	220.3 ± 1.2	217.0 ± 1.6	219.5 ± 4.5	221.5 ± 3.2
**24**	219.3 ± 2.2	220.6 ± 2.1	215.3 ± 3.9	219.0 ± 3.8	219.1 ± 5.6	220.7 ± 4.4
**48**	218.5 ± 0.8	221.0 ± 1.8	219.0 ± 1.2	220.8 ± 1.6	219.6 ± 4.6	220.7 ± 2.0
**72**	219.4 ± 1.8	223.7 ± 0.9	220.8 ± 4.3	226.3 ± 3.1	221.2 ± 5.3	221.2 ± 4.6
**96**	225.5 ± 5.9	226.3 ± 3.2	224.3 ± 8.3	223.7 ± 5.9	222.8 ± 0.3	224.6 ± 5.7
**120**	226.6 ± 3.3	226.5 ± 3.4	227.8 ± 4.6	227.2 ± 2.0	227.2 ± 1.0	229.2 ± 4.9
**144**	228.2 ± 0.9	228.5 ± 1.7	226.1 ± 3.7	233.6 ± 4.5	225.5 ± 0.1	228.4 ± 3.3

MDD: mean droplet diameter; TPN: total parenteral nutrition; Lipofundin^®^ MCT/LCT-containing TPN admixtures without (A) and with ampicillin (A + AMP). LIPIDem^®^-containing TPN admixtures without (B) and with ampicillin (B + AMP).

**Table 3:** Physical characteristics of studied TPN admixtures with vitamins and trace elements. Results are shown as the mean values of three samples ± SD.

Sample	Time	DLS		LD				Zeta Potential ± SD (mV)	pH ± SD
		MDD ± SD (nm)	PDI ± SD	Dv(90) (nm)	Particles < 500 nm (%)	Particles < 525 nm (%)	Particles > 5 µm (%)		
**A**	0	218.9 ± 3.5	0.06 ± 0.02	446	98.31	100	0	−10.90 ± 0.26	6.33 ± 0.00
	24	213.8 ± 2.9	0.07 ± 0.01	447	98.20	100	0	−11.53 ± 0.51	6.34 ± 0.00
**A + Vit + TE**	0	209.9 ± 1.1	0.04 ± 0.02	453	97.70	100	0	−12.73 ± 0.81	6.52 ± 0.00
	24	213.6 ± 2.7	0.09 ± 0.02	453	97.75	100	0	−13.57 ± 0.85	6.55 ± 0.01
**A + AMP + Vit + TE**	0	211.7 ± 2.3	0.08 ± 0.02	454	97.66	100	0	−15.47 ± 0.60	7.05 ± 0.01
	24	216.0 ± 3.5	0.08 ± 0.02	454	97.66	100	0	−15.47 ± 0.70	6.95 ± 0.01
**B**	0	219.3 ± 1.7	0.07 ± 0.02	455	98.42	100	0	−17.67 ± 1.14	6.43 ± 0.01
	24	219.7 ± 1.6	0.08 ± 0.02	456	98.18	100	0	−19.07 ± 0.71	6.43 ± 0.00
**B + Vit + TE**	0	215.4 ± 1.6	0.08 ± 0.01	457	98.74	100	0	−20.40 ± 1.05	6.47 ± 0.01
	24	218.9 ± 1.5	0.07 ± 0.03	455	98.18	100	0	−20.63 ± 0.72	6.45 ± 0.00
**B + AMP + Vit + TE**	0	213.9 ± 1.4	0.09 ± 0.01	457	98.00	100	0	−25.07 ± 0.60	7.00 ± 0.00
	24	216.2 ± 0.6	0.08 ± 0.00	457	98.74	100	0	−23.50 ± 1.28	6.88 ± 0.01

TPN: total parenteral nutrition; A: Lipofundin^®^ MCT/LCT-containing TPN admixture without ampicillin; B: LIPIDem^®^-containing TPN admixture without ampicillin; A + VIT + TE, B + VIT + TE: TPN admixtures with vitamins and trace elements; A + AMP + VIT + TE, B + AMP + VIT + TE: TPN admixtures with ampicillin, vitamins, and trace elements; MDD: mean droplet diameter; PDI: polydispersity index; DLS: dynamic light scattering; LD: laser diffraction; Dv(90): 90% of the particles lies below the value; SD: standard deviation.

**Table 4:** AMP content in TPN admixtures during storage.

Time (h)	Mean Content ± SD (%)			
	A + AMP			B + AMP
	4 ± 1 °C with Light Protection	25 ± 1 °C		4 ± 1 °C with Light Protection
		With Light Protection	Without Light Protection	
**0**	100.00 ± 0.47	100.00 ± 0.27	100.00 ± 0.57	100.00 ± 1.64
**24**	92.81 ± 0.56	90.77 ± 1.28	89.01 ± 0.85	92.97 ± 1.10
**48**	88.70 ± 1.71	76.28 ± 1.65	71.55 ± 0.46	83.02 ± 1.02
**72**	77.47 ± 1.73	71.18 ± 1.32	67.27 ± 1.84	77.20 ± 2.82
**96**	76.19 ± 1.63	67.86 ± 2.07	64.40 ± 0.73	72.82 ± 1.25
**120**	72.74 ± 1.95	62.02 ± 1.30	60.97 ± 1.68	67.41 ± 1.44
**144**	66.10 ± 1.45	60.76 ± 0.35	55.30 ± 1.86	63.52 ± 0.06

AMP: ampicillin; TPN: total parenteral nutrition; Lipofundin^®^ MCT/LCT-containing TPN admixtures without (A) and with ampicillin (A + AMP). LIPIDem^®^-containing TPN admixtures without (B) and with ampicillin (B + AMP).
